# Analysis of Genetic Variation and Potential Applications in Genome-Scale Metabolic Modeling

**DOI:** 10.3389/fbioe.2015.00013

**Published:** 2015-02-16

**Authors:** João G. R. Cardoso, Mikael Rørdam Andersen, Markus J. Herrgård, Nikolaus Sonnenschein

**Affiliations:** ^1^The Novo Nordisk Foundation Center of Biosustainability, Technical University of Denmark, Hørsholm, Denmark; ^2^Department of Systems Biology, Technical University of Denmark, Lyngby, Denmark

**Keywords:** genetic variation, SNP, next-generation sequencing, constraint-based modeling, metabolic engineering, adaptive laboratory evolution, metabolism, high-throughput analysis

## Abstract

Genetic variation is the motor of evolution and allows organisms to overcome the environmental challenges they encounter. It can be both beneficial and harmful in the process of engineering cell factories for the production of proteins and chemicals. Throughout the history of biotechnology, there have been efforts to exploit genetic variation in our favor to create strains with favorable phenotypes. Genetic variation can either be present in natural populations or it can be artificially created by mutagenesis and selection or adaptive laboratory evolution. On the other hand, unintended genetic variation during a long term production process may lead to significant economic losses and it is important to understand how to control this type of variation. With the emergence of next-generation sequencing technologies, genetic variation in microbial strains can now be determined on an unprecedented scale and resolution by re-sequencing thousands of strains systematically. In this article, we review challenges in the integration and analysis of large-scale re-sequencing data, present an extensive overview of bioinformatics methods for predicting the effects of genetic variants on protein function, and discuss approaches for interfacing existing bioinformatics approaches with genome-scale models of cellular processes in order to predict effects of sequence variation on cellular phenotypes.

## 1 Introduction

Genetic engineering has been used for several decades to manipulate microorganisms in order to allow production of valuable products, including primary metabolites (e.g., amino-acids and organic acids), secondary metabolites (e.g., antibiotics), and enzymes or other recombinant proteins (Adrio and Demain, 2010). Genetic engineering is thus a central part in the quest to establish sustainable and efficient processes for the production of fuels, chemicals, food ingredients, and pharmaceutical products.

Most of these achievements would not been possible without sequencing technologies that allowed us to identify the genetic sequences and validate the genetic manipulations in microorganisms. More recently, Next-Generation Sequencing (NGS) technologies have provided us with the capability of fast and cheap sequencing of DNA at an unprecedented scale. NGS has allowed *de novo* assembly of the genomes of thousands of organisms for which no genome sequences were previously available, ranging from complex multicellular organisms (Li et al., 2010; Nakamura et al., 2013; Pegadaraju et al., 2013; Kelley et al., 2014) to microorganisms (Soares-Castro and Santos, 2013; Yamamoto et al., 2014). NGS technologies also provide us with the means to re-sequence organisms (Atsumi et al., 2010; Wang et al., 2014), i.e., the sequencing of genetically distinct strains that are close enough to a reference strain with a sequenced genome. Re-sequencing is used to determine genetic variants ranging from single nucleotide variants (SNV) to more complex structural variants such as large deletions, inversions, and translocations. The falling cost of sequencing allows routine re-sequencing of strains isolated from the wild, monitoring the genetic stability of production strains during genetic engineering and fermentation processes, and determining the genetic basis of adaptive laboratory evolution (ALE) (Herrgård and Panagiotou, 2012). In addition to biotechnological applications, re-sequencing of microbial strains plays also a key role in other areas such as epidemiology of infectious diseases caused by bacterial and fungal pathogens, and in understanding the effects of human activity on microbial diversity and evolution in the environment.

Genome-scale metabolic models (GSMs), consisting of biochemical reactions and their relations to the genome and proteome of a cell [through gene–protein-reaction (GPR) associations], are a proven framework for the *in silico* analysis of the metabolic physiology of microbes. Genome-scale metabolic models have also been used successfully for the design of metabolically engineered strains with improved production of commercially valuable proteins and metabolites: recombinant antibodies, food additives (e.g., vanillin), organic acids, ethanol, among others (Tepper and Shlomi, 2009; Brochado et al., 2010). These models have become increasingly popular over the past decade, and more than 100 models for different organisms have been published up to this date (http://optflux.org/models). The greatest strength of GSMs lie in their simplicity and computational efficiency; new GSMs can be readily built from genomic annotations complemented with limited experimental data, and predictions from GSMs can be obtained using standard mathematical optimization methods (Varma and Palsson, 1993; Segrè et al., 2002; Shlomi et al., 2005) allowing phenotypic predictions within minutes.

Genetic variation that entails a complete loss of function – commonly referred to as gene knockout – has been successfully used to tailor GSMs to a specific genotype to improve the production of valuable compounds [e.g., biobutanol (Lee et al., 2008), sesquiterpene (Asadollahi et al., 2009), vanillin (Brochado et al., 2010), polyhydroxyalkanoates (Puchałka et al., 2008), or L-valine (Park et al., 2007)], but so far no methodological framework has been developed that would allow the incorporation of other types of genetic variants systematically. In this work, we review existing tools for analyzing genetic variants that capture more subtle changes such as synonymous and non-synonymous SNVs in coding regions or variants in promoter or other regulatory regions. We will focus on outlining the challenges of combining more subtle genetic variant information with GSMs in order to use models to predict strain-specific phenotypes.

## 2 Unveiling the Effects of Genetic Variation

### 2.1 Genetic variability

**Figure 1:**
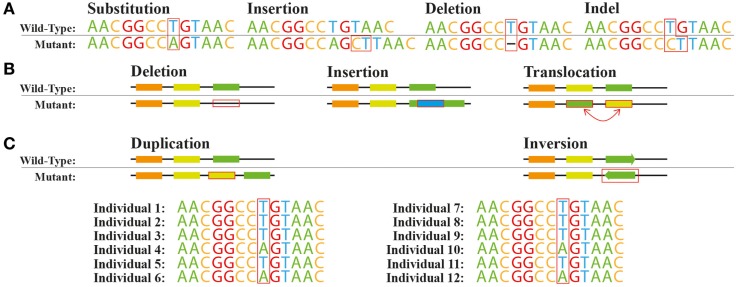
**Common genetic variations**. Variations at the **(A)** nucleotide level and **(B)** structural level. **(C)** Single nucleotide polymorphism A/T across a population.

Genetic variants, including SNVs and larger structural variants are commonly seen when natural or engineered strains are re-sequenced (Figure 1). SNVs can be found across the genome in different functional regions: (i) protein coding sequences, (ii) promoters and other regulatory elements such as ribosome binding sites, (iii) splice sites and other regions affecting transcript structures, and (iv) other genomic regions with unknown direct connections to any given protein function. Moreover, insertions or deletions of nucleotides (indels) within a coding region can cause a shift in the open reading frame usually denoted as frameshift mutations (Figure 1A). At the genome structure level, chromosomal rearrangements, e.g., swaps, inversions, deletions, and insertions, can affect the function of one or more proteins (Figure 1B).

The spectrum of the resulting effects caused by these genetic variations on individual gene or protein function or expression is very broad. Non-synonymous SNVs or in-frame indels in protein coding sequences can disrupt, enhance, or modify the activity of the protein depending on the exact amino-acid change introduced. Introduction or removal of a stop codon by specific SNVs or out-of-frame indels would be expected to result in more drastic changes of protein function. For example, the appearance of a stop codon might lead to the separation of a multi-domain protein to multiple individual single-domain proteins. The removal or replacement of a stop codon could cause translational read-through leading to an elongated protein with potential new functions (Long et al., 2003). SNVs and indels in regulatory regions such as promoters can affect the transcription or translation processes giving rise to variation in expression levels in specific proteins. In eukaryotes, variants within introns can also affect transcript structures by introducing new exons or removing existing ones. Some variations can also be completely silent with no change of phenotype, for example, a change in a stop codon location might not change the protein activity. Ideally, we should be able to predict the degree in which single and multiple genetic variants within or near a coding locus affect the relevant protein function or expression. This would allow us to rapidly make sense of the vast quantities of re-sequencing data that is becoming available without having to test the effects of all variants experimentally.

Larger-scale structural variations, such as duplications, deletions, translocations, and inversions, can have significant effects on the expression or activity of individual proteins. For example, there can be a complete loss of one or more genes, or a duplication of genomic regions can modify the expression of multiple genes within or nearby these regions (Blount et al., 2012). Very large-scale genomic changes, such as duplication of entire chromosomes, can change the activity of hundreds of proteins at once and have been reported in both natural microbial strains (Gordon et al., 2009) and in strains created by ALE (Caspeta et al., 2014). The effects of structural genomic variation are often more systemic than the effects of smaller scale variations, but any framework attempting to predict the phenotypic effects of genetic variation needs to consider both small- and large-scale variation.

### 2.2 *In silico*: Predicting the effect of genetic variants

A major challenge to understanding the phenotypic consequences of genetic variation lies in our ability to predict the mechanistic consequences of mutations. Proteins are very complex structures that fall into different functional categories and can be characterized by many distinct properties. For example, how protein activities are measured depends on their functional category: transcription factors can be characterized by their binding strength to a certain promoter region while metabolic enzymes would typically be characterized by their catalytic activity and specificity for a certain substrate. Moreover, proteins do not operate in isolation but interact with each other and with metabolites, and these interactions have consequences on the activities of proteins. Here, we provide a non-exhaustive review of the types of methods that are commonly used to predict the effects of genetic variants on protein function.

**Figure 2:**
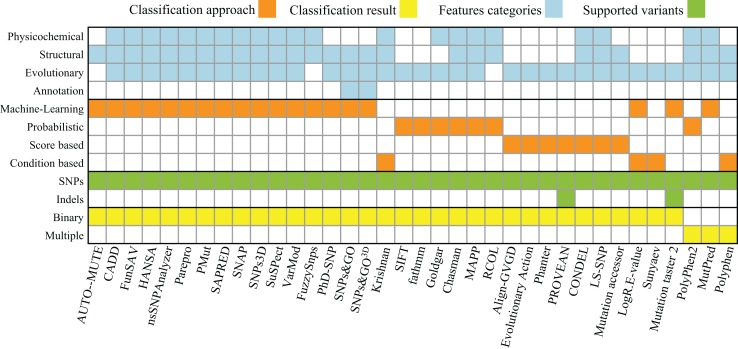
**Summary of properties and approaches for software listed in Table 1**. The approaches found fall into four different categories: *Machine-Learning*, *Probabilistic*, *Score* (calculating a summarizing score of a set of hand-picked statistics), and *Rule* (using a set of empirically derived rules). These approaches provide one of two types of classifications each: a binary classification (e.g., neutral or deleterious) or a multi-classification (e.g., benign, neutral, and deleterious). The features used by those approaches can be computed based on properties of the following five categories: (i) physicochemical properties (e.g., solvent accessibility, polarity, charge, disorder, and Grantham), (ii) structural information about the primary, secondary, and tertiary structure of a protein (e.g., α-helices, β-sheets, and coil), (iii) evolutionary properties (multiple sequence alignments, position-specific scoring matrices, and Hidden Markov models), and (iv) genome annotation (GO terms or other protein function annotations). The supported variants were determined either by accessing the tools’ websites or by the description of the approach itself.

The study of single nucleotide polymorphisms (SNP) that affect human health is one of the major focus areas of modern medical research. In human genetics, SNPs are single nucleotide substitutions found in more than 1% of a population. Several algorithms were implemented to determine the effect of SNPs, mostly specialized to the analysis of human genotyping data (see Table 1 and Figure 2). One limitation of most of these algorithms is that they are binary classifiers – deleterious or neutral, disease-causing or neutral, and tolerant or intolerant. This means that the genetic changes will either be predicted to have no effect or to cause some measurable, negative impact on the phenotype. This may not be an issue in the context of human diseases as SNP data are primarily used in diagnostics. However, fine tuning engineered microbial strains requires more than a black and white approach for predicting variant effects on protein function. This is because many genetic variants can yield proteins with either increased or decreased activity, requiring methods that are able to predict also potential gains or modifications of functions. In particular, when mutagenesis and selection or ALE methods are applied, one commonly sees gain of function mutations of specific genes that are crucial for the adaptation to, for example, new carbon sources (Conrad et al., 2011).

**Table 1:** **A summary of the available software tools for predicting the effect of the genetic variants**.

Tool	Description	Reference
AUTO-MUTE	Uses the “4-Body Statistical Potential” to compute a set of features – based on protein 3D structure – used to train a Random Forest model to predict *neutral* or *disease*-associated SNPs.	Masso and Vaisman (2010)
Align-GVGD	This algorithm is based on multiple sequence alignment and Grantham distance to identify missense SNPs. The authors propose a measure to calculate how much the substitution changes the Grantham distance.	Tavtigian (2005)
CADD	A machine-learning approach that uses a SVM model to predict deleterious phenotypes caused by SNPs.	Kircher et al. (2014)
Chasman and Adams (2001)	A probabilistic approach to identify which SNPs have an effect on the protein function using structural and evolutionary features that compare the variation against a dataset of mutations of lac repressor and T4 lysozyme.	Chasman and Adams (2001)
CONDEL	**Con**sensus **del**eteriousness provides a score computed based on the weighted average of the normalized scores of five different tools: LogR.E-value, MAPP, mutation assessor, polyphen, and STIF.	González-Pérez and López-Bigas (2011)
Evolutionary action	Evolutionary action is a function that links genotype with phenotype using evolutionary information, by quantifying the impact of SNPs on the fitness of a population; it correlates with disease-associated mutations.	Katsonis and Lichtarge (2014)
FATHMM	Uses Hidden Markov Models (HMMs) to obtain position-specific information. The prediction is based on the probability change of the HMM between wild-type and mutant.	Shihab et al. (2012)
FunSAV	A random forest classifier for predicting deleterious SNPs. It combines properties of the mutated protein with other tools (i.e., nsSNPAnalyzer, PANTHER, PhD-SNP, PolyPhen2, SIFT, and SNAP).	Wang et al. (2012)
FuzzySnps	A machine-learning approach that uses a Random Forest model trained by combining “4-Body Statistical Potential” and sequence-based features to identify tolerant and intolerant SNPs.	Barenboim et al. (2008)
Goldgar et al. (2004)	A probabilistic approach to determine if a SNP is disease-causing, which is achieved by computing the likelihood of the protein to be similar to previously classified mutated proteins in a dataset.	Goldgar et al. (2004)
HANSA	It is a machine-learning classifier that uses a SVM model to predict whether a SNP will be neutral or disease-causing.	Acharya and Nagarajaram (2011)
LogR.E-value	Uses the *E*-value computed by the HMMER algorithm using PFAM motifs to distinguish between deleterious and neutral SNPs.	Clifford et al. (2004)
LS-SNP	A workflow/database that uses predefined rules and machine-learning (SVN) approach to systematically characterize known SNPs.	Karchin et al. (2005)
Krishnan and Westhead (2003)	Two machine-learning approaches – using SVM and Decision Trees models – are used to predict the “effect” or “no-effect” of a SNP.	Krishnan and Westhead (2003)
MAPP	**M**ultivariate **A**nalysis of **P**rotein **P**olymorphism uses statistical analysis to predict the deleterious effect of SNPs.	Stone (2005)
Mutation assessor	Predicts the degree of impact in a protein by scoring the mutation based on the impact it causes regarding the properties of a multiple sequence alignment of homologous sequences.	Reva et al. (2011)
Mutation taster 2	Uses a Bayes classifier to predict disease associated effects caused by SNPs or Indels. The classifier uses a set of features that includes splicing site and polyadenylation signal information along with structural and evolutionary properties.	Schwarz et al. (2014)
MutPred	Uses a machine-learning approach to predict disease or neutral SNPs. The features used refer to a probability of loss or gain of function regarding several functional and structural properties of the encoded protein. The authors trained SVM and Random Forest models in this work.	Li et al. (2009)
nsSNPAnalyzer	Uses a Random Forest model trained with features (consisting of SIFT score and information from multiple sequence alignment and protein 3D structures) to identify disease associated SNPs.	Bao et al. (2005)
Papepro	A SVM prediction model is used by the authors to separate deleterious from neutral SNPs.	Tian et al. (2007)
Panther	Using an internal database of HMM, an evolutionary score is computed and the method predicts deleterious or neutral effects with a probability attached. The cutoff can be defined by the user (default is 3).	Thomas and Kejariwal (2004)
PhD-SNP	This approach uses one of two SVM models: one is trained using sequence profile features and the other is trained using sequence features. The choice of which model to use is based on a preliminary decision: if the mutation exists in the homology profile, the first model is used, otherwise the prediction is done using the second model.	Capriotti et al. (2006)
PMut	Predicts pathological or neutral effects of amino-acid substitutions. The prediction model is a neural network using structural-, physicochemical-, and evolutionary-based features, all calculated using sequence information only (without requiring a3D protein structure).	Ferrer-Costa et al. (2005)
Polyphen	A set of rules defined by the authors is used to predict the effect of a SNP. These rules are built based on three properties: PSIC score, substitution site properties, and substitution type properties. If one of the rules matches, the output can be deleterious or benign, otherwise the substitution is classified as neutral.	Ramensky (2002)
PolyPhen2	The follow up version of Polyphen, uses a naive Bayes predictor to predict damaging, benign, or neutral effects of SNPs. It uses structural information if available.	Adzhubei et al. (2010)
PROVEAN	**Pro**tein **V**ariation **E**ffect **AN**alyzer computes a score based on evolutionary information to predict if a genetic variant (i.e., SNP or Indel) is neutral or deleterious.	Choi et al. (2012)
RCOL	Applies a Bayes’ formula to calculate the probability of a SNP to be deleterious. The likelihood is tested using 20 structural and physicochemical parameters.	Terp et al. (2002)
SAPRED	Using a SVM prediction model, the authors combine features computed from evolutionary, structural, and physicochemical properties to predict disease associated SNPs.	Ye et al. (2007)
SIFT	Using a PSSM, SIFT determines the probability of a substitution being tolerated in a given position.	Ng and Henikoff (2001)
SNAP	Identifies non-neutral SNPs using machine-learning approaches that combines a battery of Neural Network models.	Bromberg et al. (2008)
SNPs3D	Combines a set of features obtained from protein 3D structure and evolutionary information to predict deleterious effects using a SVM model.	Yue et al. (2006)
SNPs&GO	A machine-learning approach that includes GO annotations as features in a SVM model to predict whether a SNP is neutral or disease associated.	Calabrese et al. (2009)
SNPs&GO^3D^	It is the successor of SNPs&GO. It includes new features obtained from protein 3D structure.	Capriotti and Altman (2011)
Sunyaev (2001)	This approach uses a set of seven rules empirically defined by the authors to identify nsSNPs. If one of the rules is matched, then the SNP is likely to be deleterious.	Sunyaev (2001)
SuSPect	A SVM model implementation to predict disease phenotypes caused by SNPs. The authors started with a high number of features until they identified nine that provided best performance.	Yates et al. (2014)
VarMode	A machine-learning approach using a SVN model to predict the effect of SNPs that includes information regarding known protein–protein interactions. It predicts non-synonymous SNPs.	Pappalardo and Wass (2014)

Of the existing algorithms (Table 1), *SIFT* (**S**orting **I**ntolerant **f**rom **T**olerant) (Ng and Henikoff, 2001) is often used as a gold standard to compare the performance of new algorithms or as a foundation for novel prediction strategies. SIFT and related approaches are based on the notion that evolutionary conservation can be used to predict the functional importance of each amino-acid in a protein and the impact of specific amino-acid substitutions. These methods typically use multiple sequence alignments of related proteins to determine a probabilistic description of what amino-acid substitutions are allowed in specific sites within the target protein. These descriptions can be used to determine the probability that non-synonymous coding SNPs observed in a re-sequencing data set will be tolerated by the protein; substitutions with a probability score smaller than a threshold are assumed to be deleterious (Kumar et al., 2009).

Sorting intolerant from tolerant provides only a binary deleterious/non-deleterious classification, and other methods have been developed to allow predicting cases where SNPs improve protein function. The *Polyphen* (Ramensky, 2002) and *PolyPhen2* (Adzhubei et al., 2010) approaches provide the means to discriminate three states when analyzing the effect of a SNP: benign, neutral, or deleterious. *Polyphen* uses a list of predetermined rules that combine the output of multiple algorithms using combinations of structural and sequence-based measures of mutation impact. *PolyPhen2* uses a machine-learning approach (a naive Bayes model) to predict an overall score for the variant effect, and the classification to three categories is based on thresholds. Although the algorithm is trained with human datasets, similar methods could potentially be used to build predictive models for variant effects in microorganisms. The overall variant effect score could also be exploited in more advanced methods that combine scores from different variants affecting different proteins to make phenotypic predictions.

Most studies on genetic variation focus on SNPs and disregard indels, which are also commonly observed when related microbial strains are compared to each other. The *PROVEAN* (Choi et al., 2012) and *Mutation taster 2* (Schwarz et al., 2014) approaches are capable of analyzing both SNPs and indels. *PROVEAN* uses substitution matrix scores (i.e., BLOSUM62) with gap and extension penalties to compute a variation score between the wild-type and mutant. More recently, *Mutation taster 2* computes several features (structural and evolutionary properties) for the mutated sequence using a Bayes classifier.

One possible approach for improving our ability to predict variant effects on protein function would be to predict effects of amino-acid changes on protein stability and folding (Khan and Vihinen, 2010). There are a number of tools available for these tasks (Khan and Vihinen, 2010), and stability predictions could be used to predict variant effects on protein function, as strongly destabilizing mutations would result in complete loss of function for the protein. Methods for predicting variant effects on protein stability have only been found to be moderately accurate in independent evaluation studies (Khan and Vihinen, 2010). For this reason, stability predictors should be combined with other variant effect prediction approaches to improve their predictive power for general variant effect analysis. The application of these types of stability prediction methods will be discussed in Section 3.2 in more detail together with the applications of metabolic modeling.

The majority of algorithms (53%) for variant effect prediction listed in Table 1 rely on machine-learning approaches [e.g., AUTO-MUTE (Masso and Vaisman, 2010), FunSAV (Wang et al., 2012), or HANSA (Acharya and Nagarajaram, 2011)], which is a practical strategy given the huge amount of data available for human diseases. Regarding the selection of features, most methods use evolutionary conservation information (92%) and more than half rely on structural properties (69%). The selection of sufficient features is a challenge in itself; no matter what approach is used, it is necessary to define which properties and attributes of proteins are capable of discriminating the phenotypes of interest. The improvements in the prediction capabilities provided by sequence-, evolution-, or structural-based features has been previously studied, and these studies have shown that the inclusion of structural properties leads to significant improvements in predictive power (Saunders and Baker, 2002). This has been recently confirmed by a benchmark performance test that includes several of the existing algorithms (Thusberg et al., 2011). Another effort to benchmark and improve different approaches is the Critical Assessment of Genome Interpretation (CAGI) community, which organizes a benchmark competition on predicting the effect of genetic variants on known disease phenotypes.

While the majority of algorithms aim to predict variant effects on individual proteins, a different objective is followed by the SNP-IN method that predicts how protein–protein interactions (PPIs) are affected by a SNP (Zhao et al., 2014). This is achieved by a set of features that includes the relative free energy change between wild-type and mutant PPI, the energy of all interactions in a protein complex, and other physicochemical properties, e.g., hydrophobic solvation or water bridges. Using these features, supervised and semi-supervised machine-learning approaches are used to predict how deleterious SNPs are. This approach is a very interesting, as changes in PPIs could be used to explain epistatic interactions between multiple variants. Like some previously mentioned prediction algorithms, SNP-PI requires an existing 3D model of the protein structure and, in addition, knowledge of the PPIs a given protein is involved in.

At a larger scale, genome-wide association studies are used to identify how differences between hundreds of thousands of individuals and make genotype to phenotype consequences. This approaches work as black boxes and make use of statistical and machine-learning approaches that require huge datasets. The current work and applications (e.g., clinical risk assessment) have been recently reviewed (Okser et al., 2014).

### 2.3 *In vivo*: Deep mutational scanning and Tn-seq

Next-generation sequencing has enabled studying the effects of genetic variation on individual proteins or regulatory elements *in vivo* and *in vitro*. Deep mutational scanning (DMS) is an effective high-throughput method to measure the effects of mutations on protein stability and function (Fowler and Fields, 2014). The space of all possible amino-acid substitutions in a protein is exhaustively screened by first constructing a library of sequence variants using standard techniques like error prone PCR, then by using a high-throughput assay to select variants based on a fitness measure (e.g., growth rate, ligand binding, or product fluorescence), and finally by applying deep sequencing to the selected and unselected sequence variant pools. This approach results in a matrix that contains fitness values for each amino-acid substitution discovered in the selected pool. Depending on the method used for creating sequence diversity and sequencing depth, DMS can also be used to measure epistatic effects between substitutions at different sites.

The applicability of DMS is primarily limited by the lack of high-throughput functional assays for most proteins and, so far, DMS has not been applied to metabolic enzymes. When DMS can be applied at a broader scale, the results obtained from the assay could increase the predictive power of bioinformatic tools for genetic variation analysis by providing more complete training datasets for the types of predictive methods discussed in the previous section. Methods similar to DMS can also be used to systematically study effects of genetic variation in regulatory regions on protein expression using fluorescence protein-based assays.

Here, we will highlight a few case studies using DMS and related methods to study protein or regulatory element function. In the analysis of *Saccharomyces cerevisiae* poly(A)-binding protein (Melamed et al., 2013), strong epistatic effects between substitutions at specific sites were discovered. Although epistasis was not widespread, this is worrying from a computational modeling perspective, as modeling approaches usually do not account for epistasis. Another important highlight is the identification of alternative start codons. Although analyzed in previous studies, the DMS has shown that some amino-acids can be replaced by methionine and yield functional proteins (Kim et al., 2013). This biological information can be extrapolated to other studies and is highly relevant when developing strategies to understand the effect of mutations, either *in vivo* or *in silico*. Strategies similar to DMS have also been used to systematically study the effects of variation in transcription factor binding sites and other regulatory elements such as ribosomal binding sites (Kosuri et al., 2013). These studies will build the foundation for predicting effects of non-coding sequence variants on protein expression.

The methods described above allow us to systematically study the effects of a large number of variants in individual proteins or regulatory regions. In microorganisms, it is also possible to use a next-generation sequencing-based method called Tn-seq to systematically study the effect of disruption of a large number of genomic loci on cellular phenotypes (van Opijnen and Camilli, 2013). Transposons are mobile DNA elements that can disrupt a genetic locus by integrating themselves into it (Figure 1B). Tn-seq, using high density transposon insertion libraries, can be used to interrogate the function of, for example, regulatory elements and specific protein domains in a single genome-wide assay (van Opijnen and Camilli, 2013). Tn-seq has found many applications in microbiology, and it has been used for the identification of gene function, understanding genome organization, mapping genetic interactions, or assessing gene essentiality (van Opijnen and Camilli, 2013; Yang et al., 2014). Tn-seq does not offer a resolution on the single base-pair level, but the method can be rapidly used to generate sub-gene-level information relating, for example, to the essentiality of specific domains in a protein. This information in turn could be used to improve variant effect predictions, as variants in essential domains of a protein would be more likely to be predicted to be deleterious than variants in non-essential domains of the same protein.

## 3 Predicting Phenotypes from Genotypes at the Genome-Scale

### 3.1 Statistical and network-oriented approaches for predicting phenotypes from genotypes

Section 2 focused on the task of predicting the effects of genetic variation on individual protein function or expression. However, this is only a small part of a much larger problem, which of predicting cellular or organism phenotypic effects of all the genetic variants present in a genome. This requires combing the effects of variation on the function and expression of all proteins. So far, there have been surprisingly few efforts to take all genetic variants discovered in an individual (either a human or a microbial strain) and attempt to predict how certain phenotypes would be affected by all these variants together (Burga and Lehner, 2013; Lehner, 2013).

One of the first systematic attempts toward this goal was the pioneering study by Jelier et al. in *S. cerevisiae*, where growth phenotypes of selected yeast strains under different conditions were predicted from genetic differences between a reference strain and the strain of interest (Jelier et al., 2011). This was achieved by first predicting effects of coding and regulatory variants on protein function and expression using approaches similar to the one outlined in the previous section. These variant effect predictions were then combined into a single phenotypic prediction for the strain, using published single gene deletion growth phenotyping data for a yeast reference strain under the same condition. This approach can be considered to be highly simplistic, as the effects of multiple genetic variants acting on separate proteins were treated cumulative. Despite this, the approach still allowed accurate prediction of growth phenotypes across a broad range of conditions. There have also been a number of other approaches for predicting broader phenotypic consequences of single variants by mapping the variant data onto biological networks such as PPI or genetic networks (Carter et al., 2013). However, these approaches have typically not attempted to use the whole genotype of an individual (i.e., more than one variant at a time) to predict specific phenotypes.

### 3.2 Using genome-scale metabolic models for interpreting genetic variants

The phenotype prediction methods described above are data-driven and use statistical models to predict the effects of genetic variants in the context of biological networks. However, for metabolic networks we can go beyond statistical models and graph-based descriptions to constraint-based models that are scalable to the genome-level and incorporate physicochemical, flux capacity, and reaction directionality constraints [see Price et al. (2004) for a review of constraint-based modeling]. This type of mechanistic modeling approach is very useful for understanding genetic changes that affect specific metabolic phenotypes. For example, the study of SNPs that affect mitochondrial metabolism (Jamshidi and Palsson, 2006) is a good example of how variant data can be mapped onto metabolic networks in order to explain the mechanistic basis of disease phenotypes.

A genome-scale metabolic models are composed of biochemical reactions, collected from literature and the genome annotation of an organism. This system of reactions is encoded as a matrix of stoichiometric coefficients that is usually referred to as stoichiometry matrix^1^. Assuming metabolism is in a steady-state, i.e., metabolite concentrations do not change over time, all fluxes have to balance each other. These flux-balances constitute linear constraints that can easily be analyzed using methods from linear algebra.

Furthermore, after inclusion of further constraints, e.g., known uptake and secretion rates and knowledge about reaction directionality, linear optimization methods can compute biologically relevant flux vectors that maximize defined objective functions. For example, growth can be simulated by maximizing the consumption of biomass precursors in empirically determined proportions. This type of analysis is usually referred to as flux balance analysis [FBA; see Orth et al. (2010) for a comprehensive introduction to this method].

Global optimal solutions to this linear optimization problems can be calculated very efficiently using linear programing (computation times are on a millisecond to second range for genome-scale models). Thus, one can compute thousands of phenotypes in a few minutes, simply by changing the constraints of the problem [see Lewis et al. (2012) for a comprehensive list of available *in silico* methods and (Bordbar et al., 2014) for a review of their applications].

Since the relationship between reactions, enzymes, and genes (usually referred to as GPR associations) is usually known and encoded in these models, the effect of a gene knockout can readily be mapped to the associated reactions by constraining their fluxes to be zero or by removal from the model. This way FBA can be used to compute the metabolic phenotype associated with a metabolic gene deletion, making it suitable for the analysis of genetic variation data that involves deletions or other mutations that lead to the complete loss of function of enzymes.

Flux balance analysis assumes that knockout strains can recover to an optimal growth phenotype, which might be unrealistic in cases where regulatory mechanisms – not modeled explicitly in these models – might not be able to accommodate the desired state. Other methodologies [e.g., ROOM (Shlomi et al., 2005), MoMA (Segrè et al., 2002), MiMBl (Brochado et al., 2012), and RELATCH (Kim and Reed, 2012)] employ more plausible assumptions and have been shown to improve the accuracy of knockout predictions. For example, MoMA minimizes the euclidean distance of the wild-type and mutant flux distributions, assuming that a mutant reaches the closest feasible flux distribution that is not necessarily optimal. The predictive power of FBA and these other approaches have been extensively assessed using genome-wide gene knockout assays (Snitkin et al., 2008) and transposon insertion libraries (Yang et al., 2014) and have resulted generally in a high degree of accuracy (Monk and Palsson, 2014).

Constraint-based models have also been applied to predict epistatic interactions by simulating effects of pairwise gene deletions, but with a significantly reduced accuracy in comparison to single deletions (Szappanos et al., 2011). Furthermore, simulations of multiple gene deletions have been successfully applied in developing design strategies for metabolic engineering by redirecting flux to desired products (Milne et al., 2009; Blazeck and Alper, 2010).

A number of limiting factors can diminish the ability of constraint-based models to predict phenotypic effects of loss of function mutations: (i) missing reactions and erroneous GPRs, (ii) erroneous flux constraints due to the lack of thermodynamic or regulatory information, and (iii) the assumption of a fixed biomass composition that is known to change across growth conditions. Even with these limitations, constraint-based models still outperform statistical models in predicting consequences of gene deletions (Szappanos et al., 2011).

Since constraint-based models have demonstrated good ability to predict phenotypic outcomes of single and multiple gene deletions, these models should also be useful for predicting effects of other genetic variants. A SNV or indel that is predicted to reduce the maximal flux rate of an enzyme can be used to constrain the upper bound of a flux. FBA and similar methods can be used to compute the effects of these variations on the phenotype, providing a system-wide overview of the effects caused by the substitution (Jamshidi et al., 2007). This is a fast and effective way of predicting phenotypes, but it requires that one can estimate the effect the variant has on the maximum flux rate. Nevertheless, cases of complete loss of function fall into the same category as gene knockouts, and combining the bioinformatic prediction tools discussed in Section 2.2 with modeling capabilities can be used to integrate variant data. This approach can also be extended to any number of variants and genes, with the caveat that epistatic interactions are currently not captured accurately by the models.

There is currently only a limited number of studies that use GSMs to systematically explore the effects of genetic variants on phenotypes. Chang et al. (2013) conducted a study where GSMs coupled with protein structures of metabolic enzymes (GEM-PRO^2^) were used to interpret genetic variant data of *Escherichia coli* strains evolved to tolerate high temperatures (Chang et al., 2013). In this study, a GSM of *E. coli* was constrained using experimentally or bioinformatically determined thermostabilities of metabolic enzymes. Since the maximum flux capacity of a reaction is proportional to the concentration of active enzyme, temperature changes can be modeled by varying the flux constraints accordingly. This enables the prediction of enzymatic steps that are disproportionately temperature sensitive. For the evolved strains, flux balance analysis was used to explore the adaptation of the mutated enzymes; constraints associated with mutated proteins were relaxed to explain the experimentally measured growth rates (Chang et al., 2013). The study did not include separate predictions of variant effects on protein function, but rather treated all variants observed in a protein as potentially affecting its activity.

A more recent study by Nam et al. (2014) describes the use of GSMs for understanding the metabolic effects of cancer mutations. In particular, Nam et al. use genetic mutation information, gene expression profile data, and a human GSM (Thiele et al., 2013) to construct context-specific models for different cancer types. Loss and gain of function were systematically analyzed. Loss of function was modeled as described above (i.e., constraining affected reactions’ fluxes to 0). Gain of a function, on the other hand, was modeled by adding novel promiscuous activities as predicted by chemoinformatic approaches. This approach allowed the prediction of potential oncometabolites.

### 3.3 Kinetic modeling of genetic variants

As mentioned in the previous section, constraint-based modeling does not provide any information about the dynamic behavior of a metabolic system. A full kinetic description of a biochemical reaction network can be formulated using ordinary differential equations (Heinrich and Schuster, 1996). The major advantage of using kinetic models to study effects of genetic variation lies in their ability to account for mutations affecting catalytic or regulatory sites of an enzyme, causing either a gain or loss of catalytic activity, or binding sites of allosteric regulators.

Previous studies of red blood cell metabolism provide an overview on how SNPs can alter kinetic parameters and how kinetic models can be used to explain metabolic syndromes caused by enzyme deficiencies (Jamshidi, 2002; Jamshidi and Palsson, 2009). A disadvantage of using kinetic models is that kinetic parameters are not available for most enzymes and measuring the parameters can be challenging. For this reason, building predictive genome-scale kinetic models remains a challenge (Stanford et al., 2013). Kinetic models are a viable tool for interpreting genetic variant data only in specific cases like, for example, the red blood cell that harbors a relatively simple metabolism.

## 4 Considerations and Future Directions

### 4.1 Methods and tools to predict the effect of genetic variants

Many approaches have been explored in the past decade to understand and analyze the effects of genetic variation. In particular, the most active field has been the application of NGS techniques to characterize of genetic variation in the context of human disease. The amount of disease related information makes machine-learning approaches very suitable for the purpose of predicting effects of single genetic variants. Since most prediction methods have been trained and tested with human data, many of the existing methods do not perform as well or are simply not suited for the analysis of microbial genetic variants.

The other area where the study of microbial genetic variation lags behind human genetics is the systematic collection of variant and phenotyping data. Efforts to collect human genotype and phenotype data in a standardized way are currently underway with databases such as dbSNP and European Variation Archive. The UniProt database also collects variants found in the proteins sequences when this information is available. Every day thousands of new environmental or pathogenic isolates and laboratory developed microbial strains are sequenced around the world, but there is no centralized repository for this data in common use. We argue that it is of utmost importance to collect genetic variant data together with associated phenotypic data in a standard way for microbes as well.

All the existing algorithms for variant effect prediction are used to classify variants to preassigned categories (for example deleterious or non-deleterious). The approaches that predict deleterious effects can already be handled as knockouts in modeling their phenotypic effects using GSMs, but more subtle effects of mutations are missed by this approach. In order to improve our ability to predict phenotypes, there is a need to move beyond classification toward quantitative measures of variant effects on individual protein function. There are numerous features related to protein function that may be relevant for predicting variant effects: evolutionary and conservation, physicochemical (e.g., charge, polarity, or free energy), and structural (e.g., secondary structures, spatial distances between amino-acids or B-factors).

Existing methods for predicting variant effects have been primarily focused on generic predictors for all proteins irrespective of their function (e.g., enzymes, transcription factors, transporters, chaperons, etc.) and how do they behave in their environment (i.e., interaction with other elements: proteins, metabolites, DNA, etc.). This limits the predictive power of the methods in cases where additional information is readily available such as the relatively well studied field of microbial metabolism. For example, for metabolic enzymes, information on how kinetic parameters are affected by mutations and how these parameters vary between enzymes from different species is systematically collected in databases such as BRENDA. This type of information could be used to build improved variant effect predictors specifically for metabolic enzymes.

### 4.2 Modeling and high-throughput data analysis

Improvements in genome-wide variant effect prediction can also come from improving or extending genome-scale modeling approaches. Recent innovations like GEM-PRO, as discussed in Section 3.2, fulfill the requirement of 3D protein structures to predict the effects of genetic variation at the protein level and could be used to systematically analyze the effect of genetic variation on a genome-scale for metabolism.

Approximately 10–30% of the genes encoded in a microbial genome are represented in metabolic GSMs, limiting the utility of these models for interpreting genomic variant data. Metabolic GSMs can be extended in a number of ways to increase coverage of the overall set of genes. The transcriptional regulatory network represented as interactions between transcription factors and target genes, can help extend the coverage of predictive models and can be integrated with metabolic GSMs in a number of ways (Covert et al., 2004; Chandrasekaran and Price, 2010). These integrated models have been successfully used to make phenotypic predictions.

Another recent extension of GSMs is ME-Models^3^. These models account for the entire machinery needed for gene and protein expression, providing a higher coverage of cellular functions and a higher resolution of cellular composition (O’Brien et al., 2013). ME-models have also been extended further to incorporate protein translocation from the cytoplasm to the periplasm (Liu et al., 2014). Currently, most of these extensions of GSMs have only been developed for *E. coli* and significant efforts will be required to build these extended models for other bacteria as well as eukaryotic model organisms such as *S. cerevisiae*.

The development of accurate kinetic models of metabolism, which could be useful for investigating the effects of mutations on allosteric regulation and catalytic activity, is still a tedious process. These models are usually limited to small parts of metabolism focusing on central carbon metabolism (Chassagnole et al., 2002; Peskov et al., 2012; Machado et al., 2014). There are two main reasons for these limitations: the models become huge in size and kinetic information of many enzymes is still unknown. Protocols (Stanford et al., 2013) and methodologies (Chowdhury et al., 2014) are being developed to bring kinetic modeling to the genome-scale, but the resulting models have not yet reached sufficiently mature stage for use in variant effect prediction.

In comprehensive level, a strategy for building whole-cell models by combining multiple individual models of different cellular processes including cell cycle, metabolism, transcription, and transport has been proposed (Karr et al., 2012). This strategy that also allows combining models using different representations (constraint-based, kinetic, and stochastic) was used to build a functioning whole-cell model of one of the simplest prokaryotes, *Mycoplasma genitalium*. Efforts toward building more complete genome-scale models of microbes will continue as more and more information is collected and computing power increases. These models will bring us closer to the goal of genome-wide prediction of phenotypes from genotyping data.

### 4.3 Opportunities

Genetic engineering tools, such as MAGE (Wang et al., 2009) or CRISPR/Cas9 (Xu et al., 2014), already allow us to quickly edit genomes in a precise and accurate fashion at the single base-pair resolution level at multiple loci simultaneously. These methods will allow us to map epistatic interactions of variants within a single gene and between multiple genes more comprehensively than before. On the other hand, new *in silico* tools for predicting variant effects on phenotypes outlined above open the way to a new style of modeling at the scale of single nucleotides. These new modeling tools will greatly benefit from better training datasets that can be obtained using MAGE, CRISPR/Cas9 or other genome editing methods systematically to map epistatic interactions. The application of these novel strategies provides a way to fine tune activities of proteins in the context of complete cellular networks. For example, we envision that in the future we will have predictive models of how engineering of multiple enzymes at the single amino-acid level would affect the production of a desired metabolite.

To achieve the maximum potential of genome-scale biochemical network modeling and genetic variant analysis, a link must be created between these two fields. The necessary information to connect both worlds is already there: we know the genes, the proteins, and the reactions. The major limitations are in the current methods and data sources. On the one hand, we must overcome the limitations of the tools available to predict variant effects by allowing more fine grained predictions of how a variant may affect any given protein function or expression. The usage of protein folding predictions, for example, has already been established in metabolic modeling (Chang et al., 2013), and it should be possible to use tools that predict variant effects on protein stability together with genome-scale models. On the other hand, we need to improve biochemical network modeling techniques: this is a evolving field and in the past decade there have been efforts to standardize the construction of models (Thiele and Palsson, 2010) and improving prediction methods by including high-throughput data (Machado and Herrgård, 2014).

Finally, it should be acknowledged that there will always be limitations in using solely genomic variant data as the basis for making phenotypic predictions for specific strains. We may also need to measure intermediate phenotypes such as transcript, protein, or metabolite levels for these strains in order to make predictions of how a given genotype affects a specific phenotype (Burga and Lehner, 2013). Fortunately enough comprehensive multi-omic datasets are currently being collected for wild-type microbial strains, allowing refinement of modeling and bioinformatic approaches for phenotypic prediction (Ishii et al., 2007; Skelly et al., 2013). Hopefully, systematizing such datasets and a concerted action between modelers, geneticists, microbiologists, and bioinformaticians will allow us to achieve the prediction of changed and novel metabolic capabilities of a microbial strain from genomic re-sequencing data.

## Conflict of Interest Statement

The authors declare that the research was conducted in the absence of any commercial or financial relationships that could be construed as a potential conflict of interest.

## Footnotes

^1^The rows and columns of the stoichiometry matrix correspond to metabolites and reactions respectively; negative (positive) factors represent consumption (production) of substrates (products).

^2^Genome-scale metabolic models are sometimes also referred to as GEMs.

^3^Metabolism and Expression models.
